# The evolution of universal adaptations of life is driven by universal properties of matter: energy, entropy, and interaction

**DOI:** 10.12688/f1000research.24447.3

**Published:** 2020-09-02

**Authors:** Irun R. Cohen, Assaf Marron

**Affiliations:** 1 Department of Immunology, Weizmann Institute of Science, Rehovot, 76100, Israel; 2 Department of Computer Science and Applied Mathematics, Weizmann Institute of Science, Rehovot, 76100, Israel

**Keywords:** Evolution, Energy, Entropy, Interaction, Cooperation, Niche, Fitness, Fittedness

## Abstract

The evolution of multicellular eukaryotes expresses two sorts of adaptations: local adaptations like fur or feathers, which characterize species in particular environments, and universal adaptations like microbiomes or sexual reproduction, which characterize most multicellulars in any environment. We reason that the mechanisms driving the universal adaptations of multicellulars should themselves be universal, and propose a mechanism based on properties of matter and systems: *energy*, *entropy*, and *interaction*. *Energy* from the sun, earth and beyond creates new arrangements and interactions. Metabolic networks channel some of this energy to form cooperating, interactive arrangements. *Entropy*, used here as a term for all forces that dismantle ordered structures (rather than as a physical quantity), acts as a selective force. Entropy selects for arrangements that resist it long enough to replicate, and dismantles those that do not. *Interactions*, energy-charged and dynamic, restrain entropy and enable survival and propagation of integrated living systems. This fosters *survival-of-the-fitted* – those entities that resist entropic destruction – and not only of the fittest – the entities with the greatest reproductive success. The “unit” of evolution is not a discrete entity, such as a gene, individual, or species; what evolves are collections of related interactions at multiple scales. Survival-of-the-fitted explains universal adaptations, including resident microbiomes, sexual reproduction, continuous diversification, programmed turnover, seemingly wasteful phenotypes, altruism, co-evolving environmental niches, and advancing complexity. Indeed survival-of-the-fittest may be a particular case of the survival-of-the-fitted mechanism, promoting local adaptations that express reproductive advantages in addition to resisting entropy. Survival-of-the-fitted accounts for phenomena that have been attributed to neutral evolution: in the face of entropy, there is no neutrality; all variations are challenged by ubiquitous energy and entropy, retaining those that are “fit enough”. We propose experiments to test predictions of the survival-of-the-fitted theory, and discuss implications for the wellbeing of humans and the biosphere.

## Introduction

Evolution may be defined as the transitions over time of living systems, which include cells, organisms, species and ecosystems, along with their component genes and other molecules. Evolution can take place when several conditions are fulfilled: *variations* among the evolving systems; *interactions* with the environment, both the organic and the inorganic; *destruction* of some variants and continuing *propagation* of others; and mechanisms that can *select* between the two fates. Any mechanisms proposed to drive the processes of selection and heritability would also have to explain, or at least not contradict, the reality of presently living systems; observed life constitutes a reality test for theories of evolution.

The generally accepted mechanism of evolution is *Natural Selection* resulting from *survival-of-the-fittest* ^1^. The neo-Darwinian theory of evolution has expanded Darwin’s concept of Natural Selection to include modern genetics. Natural Selection proposes that variant individuals must struggle for resources in a necessarily limited environment; the individual who manifests superior adaptive fitness to its environment wins out in the struggle to survive and procreate. The winner’s offspring inherit the winner’s genes and so enrich the species with increased frequencies of the fittest genes. Fitness, or “being fittest” is equated with reproductive success ^2^. Iteration of this process over generations eventually leads to an optimally adapted genotype for the species in the given environment ^3, 4^. Thus, species would be expected to evolve an optimum uniformity in a stable environment. Survival-of-the-fittest selection in a new environment generates a new species and restarts the optimization process.

However, the rationale behind the theory of Natural Selection has been called into question by the recent discovery that all multicellular organisms —– plants, invertebrates and vertebrates—are holobionts composed of gene products of the eukaryote organism in cooperative interaction with great numbers of resident microbiome prokaryote genes and organisms ^5^. In a word, there are no individual organisms to engage in classical Survival-of-the-Fittest struggle ^6– 8^. All multi-cellular organisms are groups.

Indeed, microbiome organisms and their genes are acquired somatically after birth and are independent of host eukaryote genome reproduction to a significant degree. Consequently, the inheritance of eukaryote genes alone cannot endow holobiont offspring with the phenotypic fitness of a progenitor holobiont. For example, a mouse that has no microbiome, a germ-free mouse, develops an abnormal gut, a crippled immune system and other abnormalities ^9^. If there be no genetically independent, procreating individuals, individual eukaryote reproductive success alone cannot be responsible for genetic progress.

Recent publications have discussed the inheritance of the microbiome ^10^ and controversy surrounds the definition of the holobiont: for example, whether the holobiont is an integrated individual or a community ^6, 11^. These matters raise questions critical to our understanding of the holobiont’s role in the process of evolution. The issue has yet to be resolved.

Another recent controversy surrounding the mechanism of evolution relates to the concept of niche construction – the co-evolution of the environmental niche with its resident species ^12^.

The theory of *Extended Evolutionary Synthesis* (EES) ^13– 15^ reconsiders evolution in the light of niche construction, the holobiont and other challenges facing classical thinking. The issue is subject to debate ^16^.

We shall not attempt to analyze or resolve these controversies; rather we draw a distinction between universal adaptations expressed by essentially all multicellular organisms and local adaptations expressed by particular species in a defined environment. This paper deals with universal adaptations of multicellulars observed in most all environments including cooperation, sexual reproduction, continuous diversification, programmed turnover and others, analyzed below. We reason that universal adaptations must have been driven and selected for survival by ubiquitous physical features of matter: *Energy*, *Entropy* and *Interaction*.

We shall first describe energy and entropy and then proceed to interaction and how their integration can account for universal adaptations. Along the way, we contrast our proposed material mechanism with the classical mechanism of survival-of-the-fittest. We term our theory *Survival-of-the-fitted*. We do not deal directly with the evolution of unicellular eukaryotes or prokaryotes; we plan to address the mechanism of unicellular evolution in a future paper.

This paper has evolved from preliminary thoughts about the adaptive immune system ^17^, the role of information in art, science and evolution ^18^ and the evolutionary relationship between information and entropy ^19^.

## Energy and entropy

### Energy is an imperative of life

Darwin did not refer to energy as a formative factor in evolution, and neo-Darwinian gene-centered discourse has paid little attention to either energy or entropy. In contrast, we shall show that energy and entropy (and networks of cooperative interaction) are more consequential to the evolution of universals than is the selfish Darwinian struggle.

Energy is defined by physicists in various ways using different mathematical formulations, each formulation suitable for a different situation ^20^. Nevertheless, we can discuss the evolutionary role of energy without having to get into the mathematical details. Dictionaries define energy as the impetus behind all motion and all activity and as the capacity to do work ^21^. Capacity to do work relates to energy in an organized or ordered form. Living systems, as we discuss below, exploit and are also damaged by energy, both ordered and disordered. The world is permeated with energy in many forms: irradiation from the sun, the earth and outer space; the force of gravity; impacts with asteroids; chemical interactions; electromagnetism; the weather—wind, rain, snow, hailstones, cold, heat; lightning; tides, rivers, floods, and droughts; fires; seismic movements and eruptions; to these inorganic elements we can add energy originating from living systems and from human technology.

### Entropy is life’s nemesis and evolution’s facilitator

The word entropy is formally defined as a measure of the number of possible microstates of a system ^22^. The term is informally used to refer to disorder *per se*, to a measurement of the amount of disorder, and to the tendency, processes or forces that spontaneously effect deterioration, destruction, or dissolution ^23^. To avoid ambiguity ^24^, we use the term to include all the circumstances and events that can lead to the destruction or dissolution of any specific arrangement.

**Figure 1.**
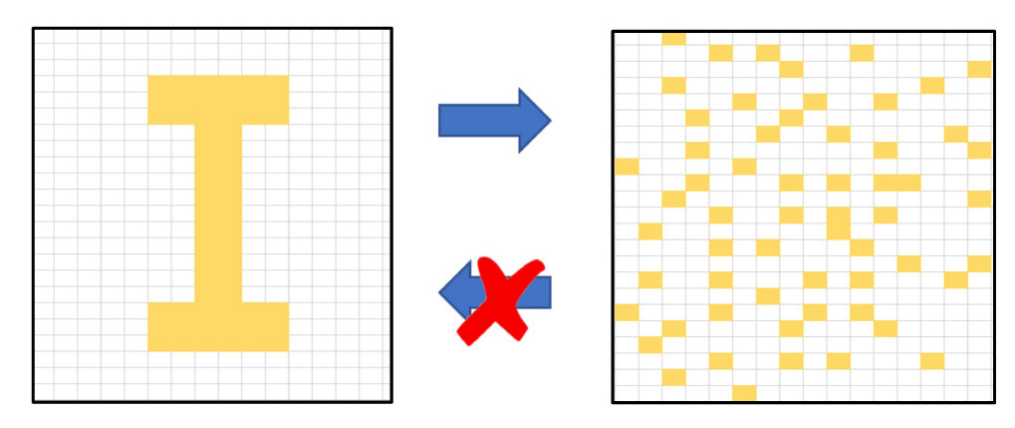
Entropy; the spontaneous dissolution of order into randomness. In this simplified conceptual representation, the border rectangles mark a closed system. The "I" structure in the left frame will spontaneously dissolve into a random disorder of its component pixels, shown in the right frame; the spontaneous process is irreversible. The challenge facing life is to resist entropic dissolution of essential structure. The figure was inspired by P. W. Atkins ^27^.

The concept of entropy derives from the second law of thermodynamics, which states that that disorder in an isolated system will continuously increase spontaneously until random homogeneity is reached; more specifically, quoting from Wehrl ^22^, “the entropy of a closed system never decreases; it can only remain constant or increase”; or, stated as the maximum entropy principle, “the entropy of a closed system in equilibrium always takes the maximal possible value”. Entropy, as we said above, is formally defined as a statistical property of any system that contains many component parts or states - so called microstates: the greater the number of parts or micro-states, the less likely will be any particular, given arrangement; randomness will prevail ( Figure 1). Moreover, the internal energy inherent in all substances ^25^ - seen, for example in Brownian motion - propels them, sooner or later, to fall apart—unless, as we discuss below, cooperative interactions and other processes hold that arrangement together or renew it.

Living systems depend on the specific arrangements and interactions of molecules, cells, organs, individuals, species, and ecosystems ^26^. Consequently, life has had to adapt to entropy, the metaphoric destroyer of specific arrangements. Ludwig Boltzmann, one of the founding fathers of thermodynamics, already made the point in 1875 ^28^:

- *The general struggle for existence of animate beings is not a struggle for raw materials – these, for organisms, are air, water and soil, all abundantly available – nor for energy which exists in plenty in any body in the form of heat, but a struggle for [negative] entropy, which becomes available through the transition of energy from the hot sun to the cold earth.*

“Negative entropy” refers to “order”.

In his book *What is Life*, the physicist Erwin Schrödinger concluded that entropy—the decay of orderly arrangements into atomic chaos—is the ultimate challenge facing living systems ^29^. Schrödinger did not attempt to relate evolution to entropy, and accepted Darwinian Natural Selection and survival-of-the-fittest as a fact (page 11). He made it clear, however, that living organisms have to pay for their order by exporting into the environment a due measure of entropy in the form of heat.

The mystery of life, says Schrödinger, is that *“existing order displays the power of maintaining itself and of producing orderly events.”* Schrödinger offers no mechanism that might maintain and produce order by restraining entropy, other than to state that *“we, no doubt, draw on experience concerning social organization and other events which involve the activity of organisms. And so it might seem that something like a vicious circle is implied.”* In other words, Schrödinger speculates that the orderly arrangements of life emerge from the order inherent in life’s ongoing activities—in a “vicious circle”; cooperative, “social” interactions somehow restrain the effects of entropy.

Despite the physical definition of entropy as a statistical measure of microstates, physicists Boltzmann and Schrödinger both relate to entropy as a concrete threat to life.

It is clear that entropy is life’s nemesis, but how can entropy also function as evolution’s facilitator? Above, we pointed out that evolution requires *variation, interaction, selection, destruction and propagation*. Obviously, entropy plays important roles in variation and destruction, but, as we shall see, entropy will also turn out to be a major factor in selection.

Over the years, many theoretical papers have been written to reconcile the evolution of life with entropy and the second law of thermodynamics (for example, see 30– 34); but, as far as we know, none of these authors have challenged the paradigmatic neo-Darwinian mechanism of survival-of-the-fittest, and none of them have invoked entropy as a selective factor in evolution, as we do here.

### Prevalent adaptations have evolved to mitigate the effects of entropy

Adaptation is often described as the aim of evolution ^35^: adaptation designates a state in which an organism or species fits the constraints and demands of its environment; to adapt is to solve a vital survival problem imposed by the environment. This concept of adaptation, however, is paradoxical; for example, wings are local adaptations for flying and fins for swimming. Local adaptations are limited to a species in a certain environment; wings and fins only solve fitness problems if one is already a bird or a fish; an elephant would only have more problems surviving with wings or fins.

Levins and Lewontin state that; *It is difficult to think of any physical force or universal physical law that represents a fixed problem to which all organisms must find a direct solution* ^36^. If you think about it, however, there is one universal physical law to which adaptation is required *a priori* for living – all creatures in any environment have to deal with entropy. Therefore, adaptations that restrain entropy are ubiquitous in living systems; extending Schrödinger, we propose that cooperative, “social” interaction is one such adaptation.

## Interactions

### Interactions fashion reality

The physicist Richard Feynman referring to the atomic and subatomic scales, claimed that “all mass is interaction” ^37^ (Prologue); similarly, Carlo Rovelli stated that all reality is interaction ^38^. Feynman suggested a sentence that bears the most information about our world for posterity: “all things are made of atoms—little particles that move around in perpetual motion, attracting each other when they are a little distance apart, but repelling upon being squeezed into one another” ^25^. Since most matter in the biosphere is made of atoms, everything made of such matter is made of moving, interacting atoms. Here we apply dynamic interaction as a building block at higher, biological scales represented by molecules, cells, organisms, species and ecosystems.

### Cooperative interactions are pervasive and central to life

We define an interaction as a relationship between two or more entities involving a transfer or exchange of matter, information and/or energy. Interactions include both struggle and cooperation: in a struggle, the participants each strive to win and dominate the others – who become the losers. In a cooperative interaction, there are no losers; the participants each gain some benefit, or at the least suffer no loss.

Living systems interact with other living systems, but they also interact with non-living matter like water, minerals and air. Interactions maintain life, and are critical to the evolution of life. Darwin proposed that evolution is driven by interactions expressed as individual struggles for survival. In a limited environment, one’s gain must be offset by others’ losses. Such a zero-sum game logically generates selfishness ^39– 41^. When, however, we look at the dominant characteristics of living systems, what we see is not only selfishness but also interactions that generate cooperation and symbiosis.

Symbiotic cooperation—living and working together—seems to have been decisive in the evolution of complex life: the primordial development of a progenitor eukaryote cell, estimated to have occurred about two billion years ago, is now thought to have resulted from the symbiotic amalgamation of two or more prokaryote (bacteria or archaea) cell types to eventually evolve a new living entity – a eukaryote cell ^42, 43^. Prokaryote life was then, and still is consummately fit; the foray of life into a complicated eukaryotic endosymbiosis is a fortuitous meander of nature that has eventually led to us complex humans and to our effects on the biosphere.

The evolution of single-celled eukaryotes into multicellular organisms is also a cooperative enterprise. The phenotype of any multi-cellular individual is the expression of at least two internal cooperative processes: First, there is the developmental and physiological cooperation of the offspring’s eukaryote genes and cells, which initially emerged from the endosymbiosis of parental germ cells. One’s eukaryote cells share a common DNA genome, but differential epigenetic expressions of this shared genome are key to interactions resulting in growth, development, differentiation, immunity, repair, reproduction, physiology, behavior, metabolism, species and ecosystems. Indeed, all processes of multi-cellular life involve cooperative interactions: all depend on a variety of cooperative interactions between molecules, cells and organisms. Even the development of a mutated, lethal tumor can progress only through cooperative interactions of the tumor cells with a microenvironment of non-tumor cells and tissues, including angiogenesis and immune system complicity ^44^.

### Cooperation includes the microbiome

The second level of eukaryote cooperative interaction is with a resident microbiome, which may include bacteria, archaea, yeasts, algae and viruses ^45^. Research is yet in an early stage, but the microbiome is clearly an essential component of all multi-cellular invertebrate, vertebrate and plant organisms, which, as we mentioned, are holobionts. The gut microbiome in humans and other organisms performs a variety of significant functions: metabolism of otherwise indigestible foodstuffs; production of vitamins and other essential molecules; detoxification of harmful substances; neutralizing or blocking pathogens; assistance in developing the immune system; warming the body; influencing brain development and behavior; and other benefits ^8^. The human species may actually benefit from the ridding of burdensome, aged humans by the timely evolution of “pathogenic” microbiome bacteria ^46^. In general, microbiome-host metabolic interactions are critical in human health and disease; an abnormal microbiome (dysbiosis) is a hazard ^47^. The microbiome dynamically changes with age, diet, gender, and other factors; for example, a change in diet can greatly expand or reduce particular components of a holobiont’s microbiome and, consequently, affect the holobiont’s health, adaptation to environmental changes and survival.

How the essential microbiome and the host organism genome are transmitted faithfully across generations is still a matter for debate ^10^. As part of this discussion, Stencel and Wloch-Salamon propose that the holobiont is not inherited as a single entity, but is a cooperating group ^11^. Hence, the discovery of the holobiont obliges evolutionary theory to reconsider group selection – a controversial idea that is incompatible with the concept of evolution driven by the heritable fitness of a single-genome individual ^48^.

### Social bonding is encoded in species genomes

Biology has recently discovered that all species of vertebrates bear genes encoding molecules like oxytocin that function to enhance cooperation, social bonding, and love, and activate brain reward centers while reducing conflict ^49^; evolution for many millions of years seems to have generated mechanisms fostering cooperation and mutual benefit. Indeed, oxytocin-like genes are borne by many invertebrates ^50^; and the products of microbiome bacteria stimulate oxytocin production in their cooperating host ^51^.

Social bonding and empathy would also appear to be programmed in the systems of mirror neurons discovered in the brains of humans and other primates ^52^; these neuronal centers are active in imitation and empathy for others ^53^. Mirror neurons probably evolved even before the primates ^54^.

Of course, mutual identification and cohesion within a group may also give rise to prejudices and to episodic conflicts with others outside the group ^55^. In any case, it is clear that evolution has generated species outfitted with innate aptitudes for bonding, cooperation, empathy and social interaction. Even the evolution of the dog from the wolf appears to have involved mutual oxytocin-related bonding with humans ^56^.

### Horizontal gene transfer is a form of cooperation

The transfer of genetic material between existing species can be seen as a further example of non-Darwinian cooperation; a species receives genes from organisms of other species that have evolved in a different environment. Horizontal gene transfer occurs regularly among prokaryotes, but also has occurred in eukaryote evolution. A notable example is the development of the placenta, a formative adaptation in mammalian evolution; placenta function involves the syncytin gene, believed to have been transferred horizontally from a virus ^57^. The survival-of-the-fittest theory, which is based on the evolution of genes internal to a single species, cannot easily account for the roles in evolution of horizontal gene transfer ^58, 59^.

### Cooperation retards entropy

**Figure 2.**
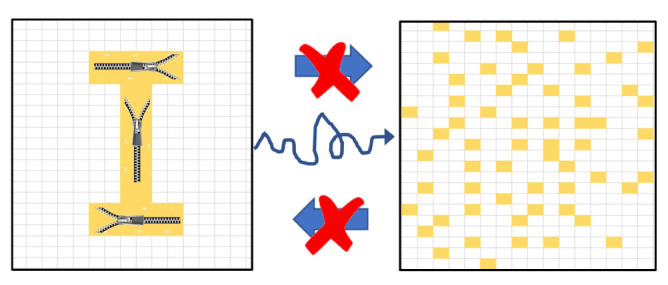
Cooperation retards entropy. Compare this conceptual figure to Figure 1. Here, the pixels that form the "I" structure on the left are held together by cooperative interactions, pictured as zippers that link the pixels. These interactions slow down the deterioration of order and structure, depicted by the long and tortuous arrow from the left to the right frame, while both short arrows are blocked.

Figure 2 illustrates that cooperative interactions between the components of an arrangement can delay the entropic destruction of the arrangement.

Darwin was aware of the intrinsic conflict between selfish struggle and cooperation ^1^ (Chapter 7); later evolutionary thinkers have attempted to explain cooperation as a result of an underlying selfish drive by theories of kinship selection or reciprocation agreements (see summary in 60), including the application of mathematical game-theory explanations ^61– 63^.

Maynard Smith and Szathmary have tabulated and studied the concept of Major Transitions in Evolution ^64, 65^. Like us, they note the importance in evolution of cooperation and sexual reproduction. However, in contrast to our entropic selection theory, Maynard Smith and Szathmary attempt to explain these and all other features of life as resulting from Darwinian competition and survival of the fittest.

Wolf, Katznelson and Koonin have identified competing interaction and “frustrated states” as a formative element in evolution and study how competition between interactions on different scales generates cooperation ^66^.

Darwinian game theory explanations for the evolution of cooperation have been widely accepted, but we believe that cooperation as a universal adaptation is more easily understandable once we hedge selfish struggle and include entropy as a selective agent in the process of evolution. An empirical example of the ability of cooperation to restrain entropic degradation is provided by the DNA molecule.

### Cooperative interactions prolong the survival of DNA

**Figure 3.**
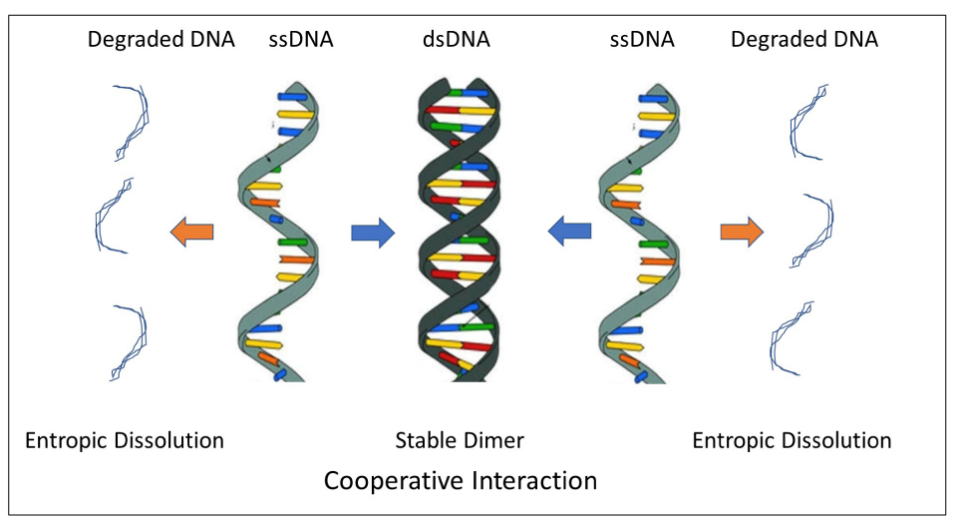
Double-stranded DNA (dsDNA), formed by the interaction between two single strands of DNA (ssDNA), resists entropy and thus enables long term survival of the composite genetic information. The colored bars protruding horizontally from the DNA strands represent non-covalent bonding sites. Interactions between the bonding sites in dsDNA protect the joint molecule from illicit, potentially damaging interactions. DNA in the single stranded form—ssDNA—is not protected from non-physiological interactions and is thereby readily degraded. Image of ssDNA and dsDNA reproduced from 75 under fair use licensing for nonprofit and educational purposes.

DNA is well known as the repository of genetic information shared by all forms of eukaryote life. Compare the fragility of single-stranded DNA (ssDNA) with the unparalleled stability of double-stranded DNA (dsDNA) ^67, 68^. The paired strands of dsDNA, bound together, are relatively resistant to random damage because each strand is in constant interaction with its sister strand; the two strands can be perceived to be in continuous cooperation; essentially all potentially reactive, non-covalent bonding energy is engaged in cooperative interactions between the strands. In marked contrast to the stable dsDNA dimer, ssDNA and single stranded RNA (ssRNA) are highly susceptible to destruction; reactive bonding energy in the single-strand configuration is not quenched by physiological interactions and is available for haphazard interactions with illicit target molecules, leading to degradation ( Figure 3). Cooperative interaction explains why stable dsDNA can be recovered from mummies or woolly mammoths thousands of years old ^69^, while unstable ssDNA and ssRNA survive for only days, at best. dsDNA illustrates how an exchange of energy between the interacting strands retards their entropic dissolution. dsDNA models the universal power of interaction to forestall entropy. We shall cite a higher scale molecular example in the section *Metabolism* below, when we discuss how dynamic metabolic interactions establish networks that restrain entropy.

### Interaction also maintains life at the macroscopic scale

We have all observed the role of cooperative interactions in maintaining life at the macroscopic scale: physical isolation or enforced bed rest of an elderly person in a hospital can lead to sudden collapse and death ^70^. Why did hospitalization kill the grandmother with the broken hip? She was receiving all the material resources needed for physical survival—food, fluids, oxygen, nursing care; what did she lack? It was not only the concentration of lethal bacteria and other such medical hazards in hospitals that killed the patient; deprivation of her customary daily activities and interactions with her familiar physical and human environments made her more susceptible to undesirable interactions ^71^. There are many observations that social interaction itself is critical. Retirement or social isolation accelerates mental and physical decline ^72^; newborns isolated from maternal interactions can sicken and die ^73^. In contrast, relationships and responses to challenge delay entropic decline and rejuvenate and maintain body and soul. Life thrives on interaction ^74^. As they say; use it or lose it.

The function of interaction in maintaining life may help us understand why some species of birds, and other organisms, have evolved to “waste” resources in elaborate courtship displays and bowers and to indiscriminately feed parasitic chicks (which may be obscenely large) of other bird species ^76^. Such behaviors clearly contradict individual Darwinian fitness. We propose, however, that seemingly wasteful activities and interactions in themselves maintain cooperative fittedness; as long as a holobiont organism or group has enough matter and energy to act, evolution will not select against a fitted action that is merely inefficient or blatantly wasteful ^77^. Interaction itself restrains entropy. Indeed, as we discuss below, living systems shield themselves from entropy, not by saving energy, but by deploying networks of energy to maintain interactions. Cooperation is its own reward.

### Prokaryotes too have evolved cooperative interactions

Microbiologists, until recently, have studied prokaryotes isolated in “pure” cultures in the laboratory; in nature, however, prokaryotes, like eukaryotes, live in complex communities of cooperating organisms in microbiomes, in biofilms, in soil, in water, and in other collective environments ^78^. Mutual group behaviors are adjusted by the exchange of molecular signals and genetic elements between prokaryotes and, in microbiomes, also between prokaryote and eukaryote cells ^79^. Cooperative interactions support the prokaryote world too.

## Metabolism

### Metabolism is essential to life

Metabolism refers to chemical interactions that produce and transform energy. The term is usually applied to the breakdown or synthesis of molecules that release, consume, or store energy.

Life has evolved metabolic arrangements that organize energy: photosynthesis and other contrivances trap disorganized light, heat and chemical energy released by our exploding sun ^80^ and our quaking earth ^81^; living systems, through metabolic networks, transform these potentially destructive forces into organized molecular and cellular structures, processes and networks of interactions that build and maintain life. Living systems also exploit energy and matter, both ordered and disordered, obtained from other living systems – creating food chains and ecosystems ^82^. The trapping of available energy, both disordered and ordered, and its conversion into networks of orderly arrangements and interactions, maintain life and its renewal in the face of entropy.

The subject of metabolism was thought to have been solved decades ago with the discoveries of the functions of vitamins and the major cycles, like the Krebs cycle, that control energy transformations. But metabolism has returned lately as a major subject of research. Metabolic pathways can differ in the substances used as fuels, the molecules consumed or synthesized, the activated enzymes, the involvement of aerobic or anaerobic pathways, the efficiency of the processes, and more. The details are beyond our present scope, but dynamic shifts in metabolic processes have been discovered not only to fuel life, but to actually signal and regulate key cellular activities and interactions. Metabolism regulates stem cell differentiation ^83^, immune cell functions ^84^, microbiome interactions ^85^, brain cell signaling ^86^, and the growth and development of cancer cells ^87^, among other functions. It is no wonder that oxytocin, the hormone of cooperation and bonding, also affects metabolism ^49^.

### Metabolism protects against entropic dissolution

The flows of energy through the networks of a living system create a unified structure that resists entropic dissolution. Building a house of cards exemplifies the power of interactive energy: a single card will not stand on a tabletop for more than an instant; two cards interacting as a triangle can stand appreciably longer—the electrostatic interactions between the cards and their balanced support keep them up, and you can progress to build a stable house of cards. That is, until you open the window and the winds of entropy blow it down. If, however, you want your house of cards to survive casual entropic winds, you only have to add a drop of glue to the interfaces between the cards. The glue augments the electrostatic bonding interactions between the cards and the house of cards will hold up, at least until the dog demolishes it. Thus do ongoing metabolic interactions (largely electrostatic) between living systems create a jointly unified structure that can restrain destructive effects of entropy.

Moreover, metabolic networks deal with energy in a step-by-step fashion—one reaction at a time; Stuart Kauffman has pointed out that such a “constrained release of energy” operates to actually reduce the entropy within the metabolic network while it increases the amount of effective work performed by the system ^33, 88^. Metabolism, besides energizing the cooperative interactions of life, acts by itself as an entropy-restraining adaptation.

An ongoing interaction between two agents reduces the numbers of random microstates — and thus of the entropy— otherwise present in each of the agents when they are not interacting. For example, as stated in 89:

- *“...A spark applied to the mixture* [of hydrogen and oxygen] *initiates a chemical reaction in which hydrogen and oxygen combine to form water. If the temperature of the system is held constant, the entropy of the system decreases because 3 moles of two differing reactants have been combined to form 2 moles of a single product. The gas now consists of a **uniform set of indistinguishable molecules**”*.

Bonding between reacting entities reduces their combined microstates and thus their total amount of entropy; the new combined entity releases entropy into the surroundings as heat.

The reduction of internal entropy achieved by integrated energy networks accounts for the evolution and persistence of interactions that would appear to detract from zero-sum Darwinian fitness. In the eyes of Natural Selection, any arrangement that efficiently saves energy should win in a struggle with arrangements that wastefully squander resources. Nevertheless, the biosphere abounds with inefficient interactions ^77^: people waste time and money on trinkets; birds, humans and other suitors invest energy on displays and rituals; butterflies, fish, birds and mammals undertake long migrations filled with peril; many species survive with energy-draining parasitisms. Only some of these “wasteful” arrangements can be explained by variants of a handicap principle ^90^, an argument that has its critics (see summary in 91).

Clearly, one can imagine mutations that could do away with such extravagances. But living systems regularly defy the logic of exclusively Darwinian bookkeeping. Cooperative interactions, like those that connect the two strands of dsDNA or a glued house of cards, can form a hedge against entropy, irrespective of efficiency or wastefulness. In other words, interaction itself resists entropy, as suggested by Schrödinger, and so Fittedness can account for arrangements and interactions that make little sense to survival-of-the-fittest thinking. Metabolism fuels the “preservative social” activities of life envisioned by Schrödinger to restrain entropy ^29^.

## Programmed renewal

### Lifespans, rates of birth, death and reproduction are programmed in species

Survival requires rebirth. The inevitable dissolution of structure implies that all livings beings are destined, in time, to fall apart and die ( Figure 1). Life has had no alternative but to effect its own renewal. In fact, we might define a living entity as one that is able to exploit energy, by way of metabolism, to renew itself ^92^.

Darwin founded Natural Selection on the assumption that living organisms strive to survive and reproduce as long and as much as they can ^1^. The fittest individuals, according to classical survival-of-the-fittest thinking, should have the most offspring. After a century and a half of refinements, qualifications, and other added nuances, the underlying principle of present-day evolutionary theory is that *“Natural selection is all about variation in reproductive success”* and *“ Natural selection is not ‘survival of the fittest,’ but rather ‘reproduction of the fittest.’* ^2^. The fittest should live the longest, provided that longevity does not compromise reproduction ^2, 93^.

But when we take a look at life as lived, we see that lifespans and birthrates are essentially programmed by an organism’s species more than by an organism’s success in struggles with competing organisms ^5, 94^. Each species has evolved its genetic endowment to encode a relatively standard lifespan for its member organisms – whether for days, seasons, or years. The apportionment of survival time, barring accidents, is fairly fixed ^93^. Classical explanations based on Natural Selection attribute fixed lifespans to a delicately balanced optimization of factors, such as age of sexual maturity, numbers of offspring, and reproductive cycle timing. But superior or successful individuals do not necessarily live the longest or beget the most offspring.

Indeed, a mindset focusing on biological survival may have delayed the recognition of programmed death ^95^ until apoptosis ^96– 99^ and autophagy ^100^ became important subjects of research.

Similar to lifespan, the time of birth and replication is closely linked to the style of life evolved by the species as a whole; the time of birth of a new organism, how long the organism lives and its numbers of offspring are associated with the way the species has evolved to make its living and manage its reproductive energy. Humans and elephants, for example, would never survive if they bore litters like rabbits; the rabbit species would never thrive if rabbits lived as long as elephants or people. Quite simply, birthrates, aging rates and spans of life are intrinsic to the genetic endowment of the species.

The human genetic disease progeria makes the point – persons bearing mutations in the LMNA gene manifest accelerated aging and usually die of “old age” in their teenage years ^101^. Standard life times are clearly demonstrated in the turnover rates of the cells that compose our bodies – different cell types manifest half-lives in tune with their healthy functions ^102^. A cell that competitively out-survives its fellows can grow into a tumor and kill the organism ^103^; unbridled survival is destructive. Death, like reproduction, cannot be left to the whims of entropy – entropy is disorganized and death by entropy, although inevitable, is too disorganized to be adaptive. A lifespan is an adaptation ^94^, not a prize for successful competition. We propose that programmed birth and death are advantageous to a species because they preempt entropic disorder by imposing order on the inevitable turnover of individuals. Death, the ultimate disorder, becomes ordered.

## Diversification, poly-determination, attractors and saltation

### Diversification, rather than uniform optimization, characterizes living systems

Replicates of any single material entity are necessarily diverse—no two genetically identical cells, for example, can occupy the same place at the same time; each cell expresses its own history and resides, as it were, in a separate environment. Beyond this fundamental, physical diversity, living systems have evolved to express higher orders of biological diversity. The concept of survival-of-the-fittest would lead one to expect that evolution should produce a single, optimal plan, approaching “perfection”, in the words of Darwin ^1^. But evolution does not produce a single optimum; evolution is marked by continuous diversification—living systems, including prokaryotes, eukaryotes, multi-cellular organisms, species and ecosystems, are composed of component parts and processes that are intrinsically variable and diverse. Diversification of genomes is evident in the tens of millions of single-nucleotide polymorphisms (SNPs) and other genetic variations found in healthy people ^104^.

Tumors express the advantages, at least for the tumor, of diversification: a tumor that includes a great diversity of clones manifests relatively more aggressiveness, greater resistance to therapy, and enhanced fittedness when metastasizing to new organs ^105^. Lethal tumors have no evolutionary future, neither for themselves nor for their hosts, but they do illustrate a short-term advantage of diversification, however selfish and ultimately self-defeating.

### Poly-determination extends diversification and retards entropy

Amazingly, living systems can utilize different combinations of their given component parts and processes to achieve a relatively uniform output. No single internal plan monopolizes a particular system. In other words, living systems can be said to be poly-determined—a given arrangement or behavior can be produced by multiple, diverse networks of interaction ^106^. A goal in football (soccer) is an accessible example of poly-determination: a scored goal is a goal, but no two are ever achieved through precisely identical field play. The poly-determination of sports is obviously an invention of human game designers; in contrast, the poly-determination of living systems has been selected by the nature of evolution.

Note that poly-determination differs from redundancy; a redundant structure or process is perceived as being a replicate of the one ideal solution; poly-determination, in contrast, is intrinsic to the multiple ways the system works to generate a given output.

A telling example of poly-determined diversification can be seen in the formation of the human species; in how many different ways has evolution been able to devise a human? We can safely assume that no two humans have ever housed identical genomes, brains, immune systems and microbiomes—monozygotic twins may emerge from a single fertilized egg, but their brains and immune systems quickly diverge and their microbiomes and epigenetic landscapes also differ. Since billions of individual holobionts have populated the human species since its inception, we can conclude that evolution has been able to concoct diverse instantiations of the human species using billions of different component recipes.

Poly-determination is also evident in the microbiome-host relationship: diverse combinations of different gut bacteria may perform similar metabolic or other functions in the host gut; in other words, a particular function may be realized by diverse combinations of microbes; as Doolittle and Booth have said: “the song is more important than the singer” ^107^.

Diversification is another name for *individuality*––no two are exactly the same; hence, evolution, as it were, has fostered individuality. Even artificially inbred mice caged together and fed identical diets manifest individual differences ^108^. Genetically identical round worms, too, manifest individuality ^109^.

Diversification, poly-determination and individuality are consequential to disease and therapy: the basic biological differences between people frustrate the traditional assumptions of Western Medicine that a given disease will appear essentially the same in different people and that an effective treatment will work in essentially all patients; actually, different subjects diagnosed with diabetes, dementia, cancer or other conditions, or people infected with a given virus or bacterial pathogen each express different clinical manifestations and will respond differently to particular treatments. Personalized medicine is a necessary consequence of entropy-selected diversification.

Biologic diversification enables living systems to keep ahead of entropy—the failure or loss of one arrangement or interaction can be compensated by alternatives. And, diversity is not a onetime thing; as we shall discuss shortly, ongoing diversification is one of the outcomes of sexual reproduction.

### A species acts as an attractor stabilized by poly-determination

Above, we noted that a stable species, like the human species, is poly-determined: the species emerges from variable dynamic interactions between its varying component organisms; different human organisms with their different microbiomes and different states of structure and activity interact to constitute a single species. From this perspective, a species can be likened metaphorically to an *attractor* ^110^; an attractor is mathematically defined as a set of number values towards which a dynamical system will tend to evolve. Similarly, despite their individual differences, the collective of organisms belonging to a given species will tend, on the whole, to exhibit a characteristic species profile; humans will be humans irrespective of their age, gender, ethnicity, and other individual differences; dogs will be dogs despite their various strains; different peach trees will bear peaches, and so forth. A poly-determined species remains stable despite the differences between its component organisms. Diversification and poly-determination endow a species with robust behavior and continuity that actually exploits entropic variation.

### Saltation emerges from diversification and poly-determination

SNPs and other diversifications intrinsic to healthy genomes suggest that single random mutations are not likely to change gene functioning; this resistance to phenotypic change indicates that one or a few mutations are not likely to affect an organism functionally. Indeed, healthy tissues tolerate large numbers of mutations ^104^; the development of a tumor, as we pointed out above, arises from critical “driver mutations” ^111^. Since healthy genomes are intrinsically diverse, a meaningful evolutionary change in a given species is also likely to withstand small genetic changes and respond primarily to a combination of many genetic changes or to a critical “driver” mutation affecting a key phenotypic character.

According to this reasoning, evolution need not advance only in small steps, as taught by Darwin, but may also progress in large jumps, or saltations ^112^. Darwin favored evolution by small increments for fear of encouraging creationist and intelligent design thinking. The roles of diversification and poly-determination in taming entropy, however, provide a physical rationale for saltatory evolution. Survival-of-the-fitted explains saltations as arising from the accumulation of multiple invisible changes that ‘finally’ exceed a certain quantum threshold; indeed, such changes can involve interactions with other organisms, materials or processes leading to entirely new entities. Phenotypical saltation poses no threat to evolution theories based on small changes.

## Sexual reproduction and diversification

### Sexual reproduction maintains the continuous diversification of species

Sexual reproduction, a defining characteristic of multi-cellular species, entails the random mixing in the offspring of the gene alleles derived from different parental organisms. Gene mixing by sexual reproduction guarantees that no multi-cellular organism will sexually transmit its exact DNA genotype to the next generation. No matter how great the Darwinian fitness of one parent, the baby will never inherit that fit genotype alone – a new baby always inherits a random mix of half the alleles of each of its parents. Sexual reproduction ensures that genetic fitness is not transmissible intact from one generation to the next. By constantly reshuffling genomes, sexual reproduction frustrates the advancement of Darwinian perfection, as assumed to result from the reproductive success of the fittest individuals ^1^.

In his book ^113^, Graham Bell asserts that *“Sex is the queen of problems in evolutionary biology”*. The problem is still open; it is clearly expressed by Burke and Bonduriansky ^114^:

- *”Why sexual reproduction is so widespread despite its substantial costs is one of the most important unsolved problems in evolutionary biology. Because sex is associated with numerous short-term costs that asexual organisms mostly avoid, theory predicts that asexual or parthenogenetic lineages should outcompete and outnumber sexual lineages, all else being equal. However, paradoxically, sex is the dominant mode of reproduction in many lineages of complex eukaryotes.”*

The community has not yet agreed on a mechanism consistent with survival-of-the-fittest struggle and the debate continues - see 115, 116. However, sex, from the perspective of fittedness, is not a problem for evolution, but a solution.

Sexual reproduction eschews the optimum, while it creates continuous innovation – we dare say that the optimal response to entropy is to avoid a single optimum. Darwin himself grappled with the problem of explaining sex ^117^. In any case, diversification is programmatically linked to the renewal of members of a species by sexual reproduction. An individual’s offspring automatically diversify the species in each generation. Quite simply, living systems are not constrained by a single “optimum” species genotype; they are programmed to avoid a single optimum; diverse arrangements retard destruction by entropy. Continuous diversification is obviously beneficial, but it is not an obvious outcome of a survival-of-the-fittest mechanism.

Of course, we must not ignore the function of sex in bonding, cooperation, family, and pleasure ^49^—evolution has programmed vertebrates like us to enjoy interactive life. Repeated sexual interactions thwart entropy even when they don’t necessarily generate renewal.

### Survival-of-the-fitted can account for same-sex sexual behavior

Sexual encounters between organisms of the same sex, widely observed in over 1,500 species of organisms, contradicts a basic assumption of Natural Selection ^115^: homosexual interactions produce no offspring; hence there is no survival-of-the-fittest mechanism for transmitting their fitness to future generations of the species. Survival-of-the-fitted, however, can explain the existence of homosexual behavior as a social interaction that enhances cooperative bonding, contributes to the environmental niche of the evolving group and restrains entropy. Thus it is not surprising that a potential for homosexuality may indeed be genetically maintained within a breeding population ^118^.

## The theory of neutral evolution

The theory of neutral evolution aims to explain the development within a species of genetically encoded traits that do not seem to make significant contributions to survival and reproduction; neutral evolutionary characters somehow are free of selection by a survival-of-the-fittest mechanism. Most neutral evolution has been described at the molecular level and includes molecular polymorphisms, variations in protein or nucleic acid sequences between organisms ^119– 121^.

Kimura proposed that the mechanism of neutral evolution was based on random mutations that did not affect protein functions; contending selectionists argued that some sort of selection must have taken place ^122^. Most evolutionary biologists today would agree with Kimura that neutral evolution results from random genetic mutation and random gene drift ^121^.

The theory of neutral evolution marks a major distinction between Darwin’s theory of Natural Selection and our theory of entropic selection: clearly, there exist genetic traits that manifest no obvious impact on individual survival and reproductive success. However, we reason that all manifestations of evolution must have undergone selection by restraint of entropy. The existence of universal entropy obliges all outcomes of evolution to undergo selection—there can be no neutrality; all persisting living systems and their component parts must withstand entropic dissolution. Evolution cannot be neutral in the face of entropy; all existing variants must, in some way, help maintain stability, or at the very least not totally sabotage stability.

## Species and ecosystems

### A species is a collective of individual organisms defined by the potential for mutual sexual reproduction

A universal outcome of evolution is the organization of organisms into higher scale species and ecosystems. Species and ecosystems are like corporations – a corporation is defined as an organization whose continuous existence is independent of the turnover of its individual members ^123^. Member organisms (and their molecules) come and go, but the species as a whole persists. Each organism is a transient subunit in the corporate body of a species and an ecosystem.

The organization of multi-cellular organisms into species is so plainly obvious that one might hesitate to enquire in public about its evolutionary origins. Darwin’s book *The Origin of Species by Natural Selection* ^1^, despite its title, did not explore the origin of species as a universal adaptation but rather the origin of the local evolution of one species into another species. The existence of species was apparently seen as an unquestionable given – just as emperors are assumed to be wearing clothes, individuals are assumed to come dressed in species. Let’s penetrate the obvious to ask what might be the evolutionary advantage of the higher scale organization of individual creatures into many different species.

The word species originates from the Latin term designating entities that look alike (from *specio* – I see). Biologists, in former times, did not know about DNA. We now use the term species in eukaryote biology to designate a collective of creatures that can breed mutually by sexual reproduction thanks to their similar DNA ^124^. Not all members of a species need to actually reproduce and they may reproduce only with certain other members of the species, and only at particular times; but they could do so at least potentially. The term species is also applied to prokaryotes, but only some prokaryotes reproduce sexually and there is no functional definition of prokaryote species; for practical purposes, similarity of 16S rDNA sequences is arbitrarily used to define bacterial species ^125^. We shall not discuss here the effect of entropic selection on the evolution of prokaryote species.

The linking of species to sexual reproduction in eukaryotes is functional; a species thwarts entropy by the continuous rebirth of its diverse members despite their inevitable death; a species, by way of sexual reproduction, also reshuffles its renewal continuously. Furthermore, sexual reproduction, by species-specific exclusivity, constitutes a barrier against invasion of the species by members of a different species; thus sexual reproduction creates a boundary, as it were, that encloses the species.

Another defining function of a species is the ability of its member organisms to construct and thrive in a shared environmental niche; the members of a species, in principle, can each perform the interactions that characterize the species – the collective of members interacts to exert an amplified effect on their shared environment. In addition, malfunctioning members of the species are replaceable.

Moreover, members of a species can specialize in particular functions: For example, male and female organisms perform different roles in social activities, group defense, habitat construction, and so forth; alpha males, for example, maintain a pack’s genetic vigor; experienced adults can educate the newborns; musically gifted persons help maintain social cohesion. So the packaging of organisms into species enables renewing, collective existence and functional similarity along with environmental continuity and individual specialization. A species can be understood as a stable attractor of diverse, continuously turning over organisms. Different species cannot reproduce across their borders, but they may link together to form ecosystems.

### Ecosystems transform entropy into order

An ecosystem is a corporation of multiple species and environments that efficiently channels flows of energy through a biosphere network ^126^. In an ecosystem, the byproducts of one species can serve as a resource for another species linked in the network. The fecal and urinary excretions of herbivores fertilize the earth and so enable the existence of all the creatures that make their living from soil or from plants; weak and dying prey animals sustain their predators, who, in turn, maintain the health of the prey species and of the environment; dead animals feed many other species along with myriads of prokaryotes who renew the flows of vital chemicals throughout the biosphere. Indeed, the dead body of one whale on the floor of the deep ocean – whale fall – can form the nucleus of an ecosystem that sustains many species including giant isopods, lobsters, shrimp, prawns, hagfish, crabs and others for decades if not for a century ^127^. See the whale as a conveyance for harvesting the ocean surface for sun-dependent forms of life and bringing, in the molecules of its dead body, the energy-rich output of the sun to the darkness of the ocean depths. Ecosystems transform sunlight and other disordered energy sources into the staff of life of the biosphere as a whole. Recall that atmospheric oxygen, essential to many forms of life, is the toxic waste of photosynthesis ^128^.

## Maintenance and repair

### Subsystems of maintenance and repair have evolved in response to entropy

Individual living cells and multi-cellular organisms, in responding to entropy, have evolved subsystems that function to repair damage inflicted by accidents, noxious parasites, injury or just plain wear-and-tear. Much of the genetic endowment of individuals and species has evolved to encode molecules and processes directed to repair. Heat shock proteins and other stress responses have been conserved since the onset of prokaryote life ^129^; DNA repair processes keep genomes healthy ^130^; immune systems of various types have evolved in all species of organisms, including prokaryotes ^131^. Life has been busy repairing itself since its beginning. We need not get into the details of these and other subsystems of maintenance; their very existence highlights the daily struggle of life with entropy.

## Environments and co-evolution

### An environmental niche emerges from a network of biological and material interactions

Any discussion of evolution must include environmental niches. The *niche* is that segment of the environment within which a species lives and interacts; the niche includes the material, biological and behavioral factors that house the species ^132, 133^. The word niche derives from the Latin *nidus*, a nest.

Classical Darwinian theory has tended to present environments as fixed arenas that provide only limited amounts of the energy, materials and space needed for survival; as a consequence, organisms are obliged to struggle amongst themselves for a livelihood ^1^. Survival-of-the-fittest, as we said, is assumed to reward the individual winners with a larger piece of the fixed environmental pie and with a reproductive advantage that disseminates their more fit genes. Darwin’s “tangled bank” of life arises, he concludes, “from the war of nature, from famine and death.” However, we see a different picture when we view the environmental niche from the perspective of fittedness: a life-sustaining environment extends beyond serving as a mere source of energy, matter and living space; an enabling environment includes interacting networks of organisms and ecosystems – effectively, the essential environment is itself a tangled web-of-life ^133^.

In contrast to Darwinian reasoning, survival does not depend merely on the ability of a cell, organism, or species to grab a maximal amount of physical energy, matter and space. Survival is not mere sustainability; survival requires that a living entity be integrated within biological and material networks that have evolved to convert entropic disorganization into organized construction. Failure to fit, or the loss of its niche, dooms the cell, organism or species to unrestrained dissolution by unrelenting entropy. Fittedness means fitting in.

### Living systems co-evolve with their niches

In contrast to classical survival-of-the-fittest thinking, successful organisms and species find or actually construct their own tailored niches. Consider the human species: the human and the chimpanzee diverged from a common great-ape ancestor about 6 or 7 million years ago ^134^. But we evolved without competing with great apes for the same environmental niche. An objective observer would conclude that we humans arose as failed apes. We simply left the apes to their natural environments and invented our own niche. The species of plants and animals that we have domesticated (wheat, maize, rice, fruits, vegetables, chickens, ducks, horses, camels, sheep, goats, and the rest) owe their success to having “found” us to serve as their environments. Deer, barn owls, raccoons, rodents, falcons, pigeons, bats, cockroaches and other feral creatures have constructed their unique niches in our belfries, homes, gardens, farms, suburbs, cities, golf courses, and more; various types of bacteria have adopted our hospitals and our bodies.

We humans evolved human culture as our niche; but niche building is not exclusive to us ^135, 136^: beavers are famous for damming lakes to build their own pools; soils are created by combined activities of prokaryotes of various types, plant roots, ants, worms, moles and other creatures. Predator and prey mutually signal each other and organize the kill to maintain both species ^137, 138^. Even bacteria build niches in particular organs in our bodies by their metabolic products and their adjustment of oxygen and acid concentrations ^139, 140^. Environment-building has been a feature of life on earth from earliest evolutionary times. Levins and Lewontin put it this way:

*It is impossible to avoid the conclusion that organisms construct every aspect of their environment themselves. They are not the passive objects of external forces, but the creators and modulators of these forces. The metaphor of adaptation must therefore be replaced by one of construction. . .* ^34^

### To live is to find or make a place in the web-of-life

If, indeed, every species fashions its own environment, we may conclude that no two species ever occupy exactly the same environment. Different species may share a physical space in land, sea or air, but each species interacts in its own unique way with that space and with its other tenants. Life converts generic “space” into a tailored “place”, a private address, as it were (see discussion in 141). In other words, the *attractor* that stabilizes a living system is not the species alone, but the species together with its co-evolving niche.

### The co-evolution of holobionts and tailored niches ensures microbiome transmission

The evolution of any species can take place only if its fittedness, selected by retarding entropy, can be passed on to future generations of offspring. The problem is that the microbiome component of a holobiont is acquired independently of host genetic reproduction; how then can the fittedness of a holobiont group be transmitted somatically to holobiont offspring? The answer, we reason, is by way of niche construction and co-evolution.

The niche of each species has co-evolved with the species in many ways, some of which enable faithful transmission of the collective of microbiome elements to the next generation—not genetically, but by a combination of somatic conditions. In plants, the seeds of a species germinate in particular types of soil suited to the species—suitable soils contain microbiomes needed by the species, within the soil itself or present in adult plants growing close to the seedlings; organisms living in water are bathed by flowing concentrations of suitable microbiomes; eggs and chicks are nested in close microbial contact with their mother birds; newborns emerging from birth canals acquire mother’s microbiomes in the course of being born ^8^.

The environmental foods that have co-evolved with resident organisms can provide needed microorganisms: humans, for example, are attracted to fermented food stuffs rich in suitable organisms such as lactobacilli, which also help preserve the food; animals transmit microorganisms when they groom and fondle each other ^142^; courting rituals and sexual interactions can transmit microbiomes; some species eat feces; attractant odor organs are usually located at oral and anal orifices; attractive odors of men and woman are produced though fermentation of secretions by skin bacteria. The reader may supply additional examples of environments and behaviors that transmit microorganisms among holobionts belonging to one or more species.

Of course, changes in the environment of a species can lead to the loss of an essential element of a species microbiome: Martin Blaser has pointed out that the overuse of antibiotics has contributed to serious pandemics of chronic diseases (diabetes, obesity, heart disease, liver disease, and others) by eliminating from the human environment critical microbiome organisms ^143^. Our discovery of antibiotics and their extensive over-use has changed for the worse the environment of the human species, and that of other species too. We are paying a high price, and, if we don’t act to characterize and preserve essential microbes, the price will only rise.

### Niches are embedded

Species construct collective niches that emerge from the individual niches constructed by the organisms that compose the species. Human culture exemplifies embedded niches: each of us (our microbiomes included) constructs our own dynamically evolving niche as we progress through life, from birth, development, education, occupation, family, and so forth. Each personal niche is fashioned with regard to collective cultural niches that precede, accompany and supersede us—language, systems of belief, values, polity, personality types, science, technologies, diet, and so forth. Niches are multi-scale fractals: niches within niches within niches. Life cannot rest at one scale; entropy must be dealt with at all habitable scales: molecular, genetic, metabolic, cellular, organismal, environmental, etc.

### Physical clocks organize biological clocks to help thwart entropy

The orderly dynamics of the physical environment impose entropy-restraining order on life. Many of the most formative arrangements and processes of living systems are organized by biological clocks – including metabolic reactions, cycles of nutrients, courting and fertility, conception, growth, development, migrations, aging, regeneration and repair, illness and death. Life is organized by recurrent time and the clocks of life are tuned to the cycles of nature manifest in the repetitive movements of the earth, moon and sun, which are expressed through cycles of precipitation, tides, seasons, temperatures, weather, dark and light, and even the frequencies of light. Living systems are also tuned to long-term manifestations of physical reality such as the movements of land masses, volcanic activities, and magnetic pole migrations. The details are far beyond the scope of this paper; we only need note that living systems dance to organized cadences of physical reality. The interactions of life have thus evolved to restrain entropic disorder by their linkage to the enduring physical clocks and order of material nature.

## Invaders and extinctions

### Species can go extinct by niche deconstruction

Evolution, we said at the outset, requires selection of some *variations* for *propagation* and others for *destruction*. At the level of the organism, propagation results from fitting into a network of supporting interactions; the destruction of the unfitted is carried out by the inexorable process of unrestrained entropy. At the level of the species, propagation is effected by construction of a co-evolving environmental niche supported by a flow of ecosystem energy. What causes the extinction of a species? Species can go extinct by the loss of their niche. For example, the dinosaurs underwent extinction when their niches were deconstructed in the wake of the earth’s collision with an extra-terrestrial body ^144^. The mammals have replaced the dinosaurs, not by struggling with them, but by constructing new niches with a variety of more effective technologies. The evolution of the organs of speech by *H. sapiens* may have enabled modern humans to replace the Neanderthals by constructing a more advanced social communications niche ^145^. Likewise, Internet shopping is replacing big-box retail stores ^146^ because Internet corporations like Amazon have evolved to exploit advanced Internet communications technology (a new mutation, as it were) to construct a new niche environment; the environments of many big-box stores are now drying up following “collision” with the Internet. Niches are found (or made) and niches are lost (or deconstructed). The loss of a niche means the loss of its species; the loss of a person’s home niche can generate a homeless person whose niche becomes a street corner.

### Invaders can destroy the niches of other species

An invader species is one that enters and thrives in a locale in which it had not previously evolved; often the success of an invading species leads to the decline or loss of other species at one time “native” to that locale. In Israel we have witnessed the spread of Grey Crows from the countryside into human neighborhoods, associated with the disappearance of the local populations of many birds including Bee-eaters; similarly, Myna birds, escaping from the cages of bird fanciers, have proliferated at the expense of house Sparrows, that themselves were invaders from England accompanying British soldiers. Perhaps the most successful invader of once natural habitats have been modern humans, who came out of Africa many tens of thousands, perhaps hundreds of thousands of years ago to take over the planet. Casting the observed events as a struggle between species followed by victory and conquest by one of them does not negate the broader view of this reality as a deconstruction of native networks of interactions in the wake of the construction of new niche interactions. Networks of interaction do not necessarily “compete” with each other, and many such networks in fact continue to coexist.

Obviously, niche deconstruction is not the only way to extinction; a species can be eradicated by a viral or bacterial pandemic, or by the dissemination of some toxic substance, or by predators, or by atomic reactions, and so forth. Entropic mishaps have no limitations.

### Humans are responsible for widespread niche deconstruction

Fittedness shoulders human culture with responsibility – we humans, who uniquely exercise conscious choice, are accountable, at least to ourselves, for our role in the state of the biosphere. Even when the human species numbered only some few millions of hunters and gatherers scattered about the earth, we appear to have eradicated many other species using only fire and weapons of stone and wood ^147^. Now we number in the billions, and are armed with ever-advancing technologies. Without intending to deconstruct the "natural" environment, our domestication of a small number of species has markedly affected the biosphere: the biomass of chickens by now far outweighs the combined biomass of all wild birds and the biomass of cattle outweighs by many fold the biomass of all other mammals combined, including us ^148^. The states of the earth and the biosphere are in our hands.

According to classical Darwinian thinking, to be fit is to win in the struggle to exploit an ever larger slice of a fixed environmental pie. The environment was viewed as a battle ground on which we, and the rest of the earth’s creatures, must fight to survive. Unfortunately, liability for undue exploitation is not inherent in the concept of Natural Selection. One may wonder whether survival-of-the-fittest thinking may have played a role in the ideology of unrestrained exploitation of the earth for economic and political “gain”. Our niche encompasses the world; unbridled “gain” will deconstruct us all.

## The emergence of complexity

Complexity is easier to recognize than it is to define. But however one defines complexity, it is evident that a biosphere that includes multi-cellular organisms is more complex than a biosphere composed of prokaryotes only ^64– 66^. Indeed, increasing complexity seems to evolve relentlessly; see for example the quote *“Everybody seems to know that complexity increases in evolution”* stated by McShea ^149^. The general increase in complexity is balanced by evidence for particular reductions in complexity, as exemplified in genome reductions discussed by Wolf and Koonin ^150^; indeed, salmon parasites show an entire loss of the mitochondrial genome and associated nuclear genes required for its transcription ^151^.

Complex species repeatedly have undergone mass extinctions ^152^. Yet evolution, as it were, picks up the simple leftovers and starts anew accumulating complexity. Why is this so? Complex systems are not more fit in Darwinian terms than are simpler systems; simple systems seem in fact to be much fitter; bacteria have survived all the extinctions suffered by the more complex organisms and will be more likely than us to survive global warming or nuclear catastrophe ^153^.

Entropic selection is compatible with the idea that complexity itself fuels more complexity ^154^. Prevailing energy and entropy, along with biological diversification, continuously drive the emergence of new mutations, combinations, interactions and functional variations. A given molecular entity will tend to increase its range of interactions over evolutionary time; the oxytocin molecule, for example, has evolved to perform a range of functions in bonding, sexual and social behavior, metabolism, inflammation and the control of stress and aggression ^49^. In each case the oxytocin molecule has added new molecular partners and so, over time, it has participated in new and more complex network interactions. Heat shock stress proteins first evolved as chaperones in primitive bacteria; they have gone on to serve as chaperones in eukaryote cells and in multi-cellular organisms, and have evolved into important signal molecules for the mammalian immune system ^155^.

Evolution seems to have created increasing complexity by adding innovations to its existing molecules, cells, organisms and species. Certain aspects of this general process have been studied under the concepts of preadaptation or exaptation ^156^. Round worms express some 20,000 genes ^157^ compared to a similar number of genes expressed in humans ^158^. These numbers are estimates subject to revision. Nevertheless, it is clear that evolution has proceeded by establishing increasingly more varied interactions and functions for a given number of genes already present in its primitive toolbox; chimpanzees and humans share more than 95% of their genes ^134^—the greater complexity of humans, expressed in language and global culture, emerges from more complex uses for common genes. Advancing complexity seems to have emerged both from novel regulatory DNA sequences and even from gene loss and not necessarily from entirely new genes ^159, 160^.

Autocatalysis of complexity is also expressed in the increased likelihood that a random variation or mutation in a gene, molecule, cell, or organism will by chance find a fit in a large landscape of different interacting entities. A complex infrastructure will tend to provide opportunities to increase its complexity; consider the ever-increasing complexity of the Internet’s evolution before our eyes. Indeed, the jump of viruses like Ebola, influenza and corona from animals into humans has resulted from the advancing complexity of human culture.

## The question of a unit of evolutionary selection

### A unit of universal selection is controversial

The designation of a "unit of evolutionary selection" is an attempt to pinpoint the primary substrate upon which the process of evolution directly acts; the unit of evolution defines the seminal entity whose evolution generates the life we see around us. Which entity is the prime mover of evolution and which entities are secondary consequences? In the beginning, Darwin proposed that individuals are the primary units driving evolution; according to Darwin the character of any species is a secondary result of the procreative success of the fittest individuals engaged in the struggle for survival ^1^. Dawkins, in contrast, argues that the gene is the primary replicator unit of evolution; individuals are merely transient phenotypic vehicles that bear the unit genes ^39^. Lewontin includes the whole spectrum of biological entities as units of evolution – genes, individuals, groups and species; according to this view, life at all its scales evolves together ^161^. Zilber-Rosenberg and Rosenberg proposed the holobiont as a unit of selection ^6^. Views and debates on the issue are reviewed by Lloyd in 162; the discussions nevertheless continue 11, 163. These designations of a unit or units of evolution assume that survival-of-the-fittest is the driving mechanism of evolutionary change; how do we define the unit of evolution from the perspective of survival-of-the-fitted?

Above, we have argued that the mechanism of universal evolution involves the interplay of three universal properties of matter: energy, entropy and interaction. These three properties exert effects on the evolution of living entities across all scales – from molecules through ecosystems. From this perspective, no single entity is “the unit” of evolutionary selection. Survival-of-the-fitted is a mechanism that makes no distinctions between any of the entities susceptible to evolution; life is the expression of its energy-dependent interactions, and all interactions must pass the tests of entropy. We would agree with positions that argue that the whole spectrum of life’s entities takes part in evolution. But we see particular entities—from cells to ecosystems—as secondary manifestations of the primary evolution of networks of interactions. If one wishes to maintain the concept of a unit of evolutionary selection, than the unit is not a particular entity like a gene or individual organism, but rather is the dynamic process of interaction itself. Indeed, the phenotype of a gene is formed by the interactions that generate gene expression, translation into proteins, post-translational modifications and all the rest. The organism, too, is a dynamic product of all its internal and external interactions. We may perceive entities, but all are snapshots of dynamic interactions. The progenitor of evolution is interaction.

## Theories of evolution influence human behavior

### The concept of survival-of-the-fittest has been misused to justify cultural domination

Whether or not Natural Selection is a “law of nature” is controversial among biologists ^164, 165^; nevertheless, extreme segments of Western Culture have usurped survival-of-the-fittest to justify an ideology of conquest and dominance. Lamentably, Natural Selection, at one time or another, has been invoked to justify colonialism, racial purity, eugenics, genetic cleansing, dictatorships, unfettered capitalism, unequal rights, and other harsh dissonances (see, for example, 166, 167). Obviously, survival-of-the-fittest is a scientific theory and not a benchmark for human behavior; nevertheless, Natural Selection has been hijacked by agendas of exploitation.

Updating the mechanism of evolution, as we have done here, to highlight the roles of *cooperation, interaction* and *diversity* throughout biology might serve to encourage rational human behavior in the realms of governance, economics and social justice.

### How can we reconcile the holobiont human with the individual human?

The discovery that all multi-cellular organisms are holobionts raises a paradox; at the biological level, there are no individuals; living systems, nevertheless, do manifest group individuality. Yet, the psychological core of each human is his or her feeling of an exclusive existence expressing private thoughts, feelings, and a specific history of being and relationship. Our internal perception of uniqueness is confirmed independently by our individual social and legal responsibilities. Can we resolve the seeming contradiction between our realities as both biological groups and functioning individuals?

Look at it this way: our psychological and institutional individuality, like our body, is created by group interactions and relationships, not merely with microbes, but with the people and the world in which we reside; our identity is the outcome of our history of bonding with others. Echoing Levins and Lewontin ^36^, we construct our niche by the nature of our interactions—we and our worlds co-evolve. We achieve a self by constructing a shared, interactive environment. Thy neighbor is part of thy evolving self.

## Reconciling survival-of-the-fitted with Darwinian survival-of-(only)-the-fittest

Physicists point out that life, like all the rest of the material universe, must accommodate the Second Law of Thermodynamics and the phenomenon of entropy ^27, 29^. Here, we have reasoned that the evolution of life, a dynamic expression of life itself, must also accommodate entropy. Consequently, stable outcomes of the evolutionary process must provide some solution to the spontaneous dissolution of order; entropy, in one way or another, must act as a selective factor in evolution – it would not be reasonable to conclude otherwise.

What about the concept of Natural Selection, a cornerstone of modern biology? One may ask, why not conclude from the above discussion that entropic selection also serves survival-of-the-fittest, without invoking survival-of-the-fitted. In other words, perhaps Natural Selection would select, among others, those individuals who counteract entropy most efficiently and, as a result, leave more progeny than others. This, however, does not work for at least four reasons: as we have discussed above, (i) there is no competition in dealing with entropy; a successful individual or species need not retard entropy most efficiently or resist entropy better than others, but only fit in sufficiently with a supportive network of interactions; (ii) not only the one most reproductively successful variant survives, but many others readily find their niches; (iii) many obviously advantageous features are not rewarded with reproductive advantage; and (iv) increased reproduction is at times more of a problem than a desired outcome.

It is conceivable that different manifestations of evolution might be assigned to a combination of fitness (Natural Selection) and fittedness (Entropic Selection). Entropy would seem to account for the more universal adaptations of evolution we have discussed here. In contrast, Natural Selection might drive more local, species-restricted adaptations. Certainly, the quantitative assessment of each mechanism would be different: the advance of evolution by individual fitness would require measurement of the birth rates of generations of descendants of particular test individuals – a difficult, if not impossible task; fittedness based on the strength of ongoing cooperative interactions could be measured, at least at the molecular level, by the amount of energy needed to disrupt the interaction: For example, the strength of the interactions that hold together dsDNA strands is reflected in the amount of heat that has to be applied to disrupt the interaction, as in the PCR reaction ^168^. In any case, a fresh look at the mechanisms of evolution is in order.

## Analysis and modeling

A theory can be invoked to explain any phenomenon; a theory is scientific when it proposes a platform for continued rational analysis and when it can support testable predictions. How may we proceed to analyze survival-of-the-fitted?

### Fittedness can be analyzed using tools of systems biology

Survival-of-the-fitted fits generally into the realm of systems biology, including dynamic complex systems and their attractor states. The perspective and the methods of systems biology go beyond a linear chain of discrete causes and effects; advancing information technologies have extended the scope of biology research, enabling analyses of complex interactions that generate dynamic self-organizing systems. “Big data” are collected, defined and analyzed for associations involving networks, modules, emergent properties and other organizational structures and principles. Systems’ understanding emerges from simulations and mathematical models ^169, 170^.

Such modeling could build upon the known generation of orderly structures from chaotic thermodynamic behavior at non-equilibrium states, at multiple scales (see, for example Turing ^171^, Prigogine ^172^, Kauffman ^33^, Jimenez ^173^, and England ^174^). We plan to model how such erratic structures will survive longer if they chance to fit into supporting interactions with existing structures; this mechanism could well provide novel arrangements for advancing evolution.

Stuart Kauffman has modeled the emergence of life and the biosphere using the concepts of living organisms as *autonomous agents* who navigate *fitness landscapes* to *make their living* with the aid of genetic *code scripts* ^33, 88^. These concepts well suit a survival-of-the-fittest mechanism. However, our proposed evolutionary mechanism of entropic selection of holobionts and cooperative interactions for fittedness does not require the concepts of autonomous agents, fitness landscapes and gene-centered competitive success. Our nascent models are likely to differ from those considered in the past. In any case, the experiments of evolution have been and are being done by nature, analyses of the results need human computation. A system’s biology of evolution is in the offing.

### Predictions

Here, we propose some experiments and predictions, not all of which are currently feasible, to test survival-of-the-fitted:

1. Germ-free mice or rats are raised behind barriers and deprived of gut microbiomes, but they can be reconstituted by feeding them with defined clones of bacteria; one group could be fed with a single monoclonal gut bacterium and other groups could be fed with combinations of diverse bacterial clones. The survival-of-the fittest paradigm would predict that the microbiomes developing in the groups of mice fed with diverse clones of bacteria would evolve over time into microbiomes populated with only a single clone of the fittest bacterium. The microbiomes in the animals fed with a single clone, in the absence of competition, would not evolve.
  In contrast, the survival-of-the-fitted paradigm would predict that the microbiomes of the animals fed only with a single monoclonal species of bacteria would in time respond to entropy to evolve into a diversity of bacteria expressing different genes. The animals fed a diversity of bacteria would continue to express a diverse microbiome. Thus, the contending *fitness and fittedness* theories of evolution would each lead to contradicting predictions.
2. Evolution will take place in the absence of competition and in environments with unlimited resources; contrary to Darwinian teachings, a struggle for fitness is not required because entropy prevails in the best of worlds. Diversification can be tested under controlled experiments *in vitro* ^35, 175^. Monoclonal, initially invariant yeast, bacteria or *C. elegans* could be grown under conditions of “optimal” nutrition and density in monitored cultures and observed for the evolution of genetic and phenotypic diversification in the absence of struggle.
3. Systems that foster diversity and individuality will thrive better than systems limited to an “optimal” archetype; cooperation and symbiosis will emerge in diversified collectives. This prediction can be tested today in a variety of systems at various scales, from single cells to societies and cultures.
4. Sexual reproduction (or an equivalent system of reproducing, programmed diversification) will evolve in multi-cellular living systems. Testing this might require the development of novel *in vitro* culture systems.
5. Complexity will increase automatically, even in an *in vitro* culture of initially uniform populations. Observations of new interactions could be made using long term culture systems.
6. Life on other planets will be seen to have evolved, provided that entropy holds sway there.
7. All living systems anywhere will evolve programmed renewal.

Predictions # 6 and # 7 will require the development of suitable probes of habitable planets distant from earth. Perhaps extraterrestrial life may one day be studied at a distance by telemetric analysis of metabolism or other manifestations of life.

Finally, we would predict that the well-being of the biosphere will be aided by realizing intelligent interactive cooperation and shunning selfish struggle. This prediction is now being tested by observing our changing world, obviously without a control group. Let us each work to fulfill this prediction.

## Data availability

No data is associated with this work.
