# Supplementary material for: The evolution of universal adaptations of life is driven by universal properties of matter: energy, entropy, and interaction
Source: F1000Res. 2020 Sep 2;9:626. Originally published 2020 Jun 18. [Version 3] doi: 10.12688/f1000research.24447.3 (PMC7416572; doi:10.12688/f1000research.24447.3)
Supplement: Peer review history

# Peer review: Reviewer response for version 2

**DOI:** 10.5256/f1000research.28164.r67392

**Published:** 2020-08-10

**Authors:** Susan Stepney

**Affiliations:** 1 Department of Computer Science, University of York, York, UK

Cohen and Marron discuss a view of evolution in the context of entropy: that the main driver underpinning change and evolution at all scales in living organisms is the need to resist the inevitable decay of that organisation, the need to resist the increase in entropy.  They provide extensive examples of the benefits of organisation, how there are no isolated organisms, and how viewing biological systems, from cells to ecosystems, through the lens of interactions allows understanding of the existence of various universals in biology.  They conclude by suggesting investigations to test this view.

 I find the argument compelling and thought-provoking as it stands.  However, there are a few places where I believe links to other work in literature could help further demonstrate both the novelty of the approach, with its focus on evolution specifically, and its connection to other thermodynamic takes on biology.

 In particular I think it would be useful to include a little on the work of Kaufmann (constrained release of energy; non-equilibrium flow into the adjacent possible; fourth law of thermodynamics; etc), and of Schneider & Sagan (open systems and non-equilibrium thermodynamics; life reducing energy gradients and thereby *accelerating* the increase in entropy; etc).

- Stuart A. Kauffman. *Investigations*. Oxford University Press. 2000
- Eric D. Schneider, Dorion Sagan. *Into the Cool: energy flow, thermodynamics, and life*.  University of Chicago Press. 2005

Also, prediction #2 would benefit from a comparison with Lenski’s long term evolution experiment.

 There are also places where the terminology and argument could be tightened up somewhat:

1. p3: although there are clues in the subsequent text, it might be useful to add a couple of sentences saying explicitly why the evolution of unicellular organisms is not dealt with.
2. p4: the statement of the 2 ^nd^ law would better read: *disorder in an isolated system does not decrease*.  (In thermodynamics, a *closed* system can still exchange energy with its surroundings, eg a constant temperature heat bath, whereas an *isolated* system cannot.  Also, entropy does not continue to increase past thermodynamic equilibrium.)
3. p5, col2: “everything made of matter is made of … atoms”; this is true only under certain physical conditions: the sun, for example, is a plasma; a neutron star is made of degenerate matter. 
4. p8, col2: “Bonding between reacting entities reduces … the total amount of entropy.”  It reduces the entropy of the atoms, but does not reduce in total: the rest is carried away by energy released by the exothermic reaction and absorbed by the constant temperature heat bath.
5. p9, col1: “define a living entity as one that is able to exploit energy, by way of metabolism, to renew itself.” The autopoiesis work of Maturana and Varela could be referenced here.
6. p13, col1: deer, barn owls, etc, co-existing with human environments, are called “feral” creatures.  Feral rather means “living wild, having escaped from captivity or domestication” (as with the Mynah birds on p14); here a better term would be “synanthropic”.
7. p13, co1: all the examples of animals constructing niches: bird nests fit here too.
8. p13, col1: “each species interacts in its own unique way with that space”: this is reminiscent of the William James quotation: “Other sculptors, other statues from the same stone! Other minds, other worlds from the same monotonous and inexpressive chaos!
9. p14, col2: on the biomass of domesticated species, the point could be made even more dramatically by saying: “the biomass of livestock outweighs by many fold the biomass of all wild mammals combined, and even outweighs the biomass of humans”.  If “survival of the fittest” holds, cattle are fitter than humans!
10. p17, prediction 6: “provided that entropy holds sway there”; why would entropy *not* hold sway there?  All the arguments in this paper assume 2 ^nd^ Law inescapability.

There are also a couple of typos.

1. p3, revision box, point 4: Szathmary’s name is misspelled.
2. p15, line 1: repeated “by”.

Is the topic of the opinion article discussed accurately in the context of the current literature?

Yes

Are arguments sufficiently supported by evidence from the published literature?

Yes

Are all factual statements correct and adequately supported by citations?

Yes

Are the conclusions drawn balanced and justified on the basis of the presented arguments?

Yes

Reviewer Expertise:

Complex systems, artificial life

I confirm that I have read this submission and believe that I have an appropriate level of expertise to confirm that it is of an acceptable scientific standard.

# Response

**Published:** 2020-08-25

**Authors:** Assaf Marron

**Affiliations:** Weizmann Institute of Science, Israel

We thank Susan Stepney for her expert review, positive comments, and valuable suggestions, and for drawing our attention to additional relevant publications.

Version 3 is now amended to address many of the comments and suggestions. Specifically: 

1.    In the introduction, we stated that we plan to address the mechanism of unicellular evolution in a future paper.

2.    In the section “Entropy is life’s nemesis and evolution’s facilitator”, we edited our statement of the second law of thermodynamics, and added published recitations of two other brief statements of the law, all of which address the comment that entropy does not always increase.

3.    In the above section, we added to the list of papers exploring the connections between evolution and entropy citations of "Investigations" by Kauffman and "Into the cool: Energy flow, thermodynamics, and life" by Schneider and Sagan. We have elaborated on the comparison with Kauffman: In the section “Analysis and modeling,” A key point in this comparison is the fact that our proposed evolutionary mechanism of entropic selection of holobionts and cooperative interactions for fittedness does not require the concepts of autonomous agents, fitness landscapes, and gene-centered competitive success. Our nascent models are likely to differ from those considered in the past. 

4.    In the section “Interactions”, we reordered and rephrased the sentences to clarify and add context to Feynman’s quote that “everything is made of atoms”. 

5.    In the section “programmed renewal,” we cited the work of Maturana and Varela on autopoiesis and the definition of living systems. 

6.    In the section “Metabolism protects against entropic dissolution,” we added citations to Kauffman’s discussion of the constrained release of energy. 

7.    In the above section, we clarified that bonding releases entropy into the environment as heat.

8.    In the section “Humans are responsible for widespread niche deconstruction”, we explicitly included humans in the discussion of animal biomass.

9.    We kept our use of the word “feral” in the sense of “not domesticated”, in line with the main definition of the OED and meaning 2a in Merriam-Webster (though both also include the narrower meaning offered in the review).

10.    We agree with Susan Stepney’s association to William James inspiring quotation, but decided not to add it to the paper. 

11.    We kept the cautious conditional regarding planets in other galaxies “provided that entropy holds sway there”

12.     We corrected several typos.

We thank Susan Stepney again for the detailed and valuable review. 

Irun R. Cohen and Assaf Marron

# Peer review: Reviewer response for version 2

**DOI:** 10.5256/f1000research.28164.r68397

**Published:** 2020-08-07

**Authors:** Eugene V. Koonin

**Affiliations:** 1 National Center for Biotechnology Information (NCBI), National Library of Medicine (NLM), National Institutes of Health (NIH), Bethesda, MD, USA

I appreciate Cohen's and Marron's response to my review of this paper. The major issues that I raised have been addressed, in the least, by siting the appropriate literature. We still do not see eye to eye with regard to 'survival of the fitted' vs 'survival of the fittest', where I fail to recognize a substantial distinction. However, the authors are entitled to their position.

Is the topic of the opinion article discussed accurately in the context of the current literature?

No

Are arguments sufficiently supported by evidence from the published literature?

No

Are all factual statements correct and adequately supported by citations?

No

Are the conclusions drawn balanced and justified on the basis of the presented arguments?

Partly

Reviewer Expertise:

Evolutionary biology

I confirm that I have read this submission and believe that I have an appropriate level of expertise to confirm that it is of an acceptable scientific standard.

# Peer review: Reviewer response for version 1

**DOI:** 10.5256/f1000research.26968.r65123

**Published:** 2020-07-13

**Authors:** Eugene V. Koonin

**Affiliations:** 1 National Center for Biotechnology Information (NCBI), National Library of Medicine (NLM), National Institutes of Health (NIH), Bethesda, MD, USA

Cohen and Marron present a concept of evolution that centers on interactions on different level - molecular, cellular, organismal etc. - which are perceived as a universal anti-entropic force. Entropy is understood as a tendency for increasing disorder and is viewed as the principal selective factor in evolution. The antientropic evolution is postulated to be 'universal', hence no neutral evolutionary processes. The authors, then, forward the principle of 'survival of the fitted' whereby all biological entities that counter entropy with sufficient success to remain in an ordered state survive.

 Although not exactly new (indeed, it goes back to Boltzmann who the authors duly cite!), the notion of the fundamental nature of the antientropic tendencies in evolution is worth putting forward. More specifically, the emphasis on interactions permeating the entire biological organization as a universal antientropic factor is worthy and might appeal to many biologists as a fresh perspective eon the general character of evolution..

 However, I find several areas within and around these where concepts where I feel compelled to mount criticisms, and not altogether minor ones. 

 First, to me, the 'survival of the fitted' appears to be a non sequitur. Let us say, everything is fine with entropy as a selective factor and interactions, but why not survival of the fittest, that is, those that counteract entropy most efficiently and, as a result, leave more progeny than others?

 Second, in all their discussion of entropy, the authors refer only to classical,equilibirum thermodynamics. As far as I can see, this discussion cannot be complete without taking into account the concepts of non-equilibrium thermodynamics, in particular, maximization of entropy production and dissipative structures. The literature on these subjects is extensive, starting with Prigogine.

 Third, to me, it is inexplicable how and why, when discussing the pivotal roles of interactions at different levels in evolution, the authors fail to include Maynard Smith-Szathmary's concept of major transitions in evolution (Ref. 1 ^1^ and references therein) and, especially, the generalized form of this concept that comes uncannily  close to the concept of interactions-driven evolution discussed here ^2^.

 Fourth, I believe that, along with the key role of interaction, it is essential for developing a general concept of evolution, to consider competition between interactions on different scales and the resulting frustrated states ^2^.

 Finally, more with respect to the presentation of the material. The figures in the current manuscript are over-simplified and, overall, underwhelming. Figure 1 is a trivial illustration of the concept of entropy and is superfluous (readers unfamiliar with the second law will not benefit from reading this article) whereas for Figures 2 and 3, more elaborate schemes are advisable.

Is the topic of the opinion article discussed accurately in the context of the current literature?

No

Are arguments sufficiently supported by evidence from the published literature?

No

Are all factual statements correct and adequately supported by citations?

No

Are the conclusions drawn balanced and justified on the basis of the presented arguments?

Partly

Reviewer Expertise:

Evolutionary biology

I confirm that I have read this submission and believe that I have an appropriate level of expertise to confirm that it is of an acceptable scientific standard, however I have significant reservations, as outlined above.

## References

[1] : Toward major evolutionary transitions theory 2.0. *Proc Natl Acad Sci U S A*.2015;112(33) : 10.1073/pnas.1421398112 10104-11 10.1073/pnas.1421398112 25838283PMC4547294

[2] : Physical foundations of biological complexity. *Proceedings of the National Academy of Sciences*.2018;115(37) : 10.1073/pnas.1807890115 E8678-E8687 10.1073/pnas.1807890115 PMC614047030150417

# Response

**Published:** 2020-07-21

**Authors:** Assaf Marron

**Affiliations:** Weizmann Institute of Science, Israel

We appreciate Eugene Koonin’s positive opening remarks and his expert review and suggestions for improving the paper.

 The specific recommendations for improvement are five:

 First, “everything is fine with entropy as a selective factor…but why not survival of the fittest, that is, those that counteract entropy most efficiently and, as a result, leave more progeny than others?”

 This is an important question and we have addressed it directly in the revision.  Briefly, we reason that dealing with entropy does not directly require competition and struggle; one need only to fit in “sufficiently”; moreover, variants that are not “the fittest” readily find other niches, and many advantageous phenotypes are not necessarily associated with reproductive advantage; indeed, increased reproduction is at times more of a problem than a desired outcome. This is, of course, in addition to the distinction the paper makes between universal adaptations driven by entropic selection and local adaptations, which may well be based on reproductive advantage and Darwinian natural selection.  See the section titled “Reconciling survival-of-the-fitted with Darwinian survival-of-(only)-the-fittest”.

 Second, “the authors refer only to classical, equilibrium thermodynamics.” We appreciate the reference to the work of Prigogine and others on the emergence of order in multiple scales from chaotic thermodynamic behavior both in equilibrium and non-equilibrium states.  In our revision, we have added the suggested references and others as important elements in quantitative analysis, simulation, and prediction. See the section titled “Analysis and modeling”.

 We thank Eugene Koonin for calling our attention to these ideas.

 Third, the first draft of the paper neglected to cite the concepts of Maynard Smith and Szathmary on Major Transitions in Evolution. Our revision now refers to this work which, among others, highlights the pivotal roles of interaction and the relentless increase of complexity. See subsection “Cooperation retards entropy” in section “Interactions”.

 Fourth, the Reviewer suggested that the paper should “consider competition between interactions on different scales and the resulting frustrated states” as a formative element in cooperative interaction. In the revised paper, we now incorporate this idea and cite the relevant paper by Wolf, Katznelson and Koonin. See subsection “Cooperation retards entropy” in section “Interactions”.

 We also added references to Maynard Smith & Szathmary and to Wolf, Katznelson and Koonin in the section titled “the emergence of complexity”.

 Fifth and finally, the Reviewer suggested that the Figures are over-simplified and superfluous (Figure 1) and “for Figures 2 and 3, more elaborate schemes are advisable.” In the revised paper, the captions clarify that the Figures are meant to serve only as simplified conceptual anchors or mnemonics for the principles of the theory.

 We thank Eugene Koonin for raising these points which we feel have improved the paper considerably.

 Irun R. Cohen and Assaf Marron

# Peer review: Reviewer response for version 1

**DOI:** 10.5256/f1000research.26968.r65121

**Published:** 2020-06-23

**Authors:** Eugene Rosenberg

**Affiliations:** 1 Department of Molecular Microbiology and Biotechnology, Tel Aviv University (TAU), Tel Aviv, Israel

Cohen and Marron offer a highly original concept of evolution, a subject fundamental to biology and philosophy. They begin by providing a clear and well-written description of neo-Darwinism, recent extended theories of evolution, which take into account epigenetics and microbiomes, and entropy. They then posit their main point- that the evolution of complexity is driven by cooperation, which is a mechanism to avoid entropic destruction. They argue that this cooperation is required at all biological levels, from molecules to ecosystems. For example, they point out the cooperation of individual strands of DNA to form double-stranded DNA helps avoid entropic destruction.

 Like most revolutionary ideas in science, the evolution of universal adaptations of life driven by the properties of matter: energy, entropy, and interactions, will be difficult for evolutionary biologists to accept. However, this article should be read and seriously considered by biologists. The authors end the article by suggesting some interesting experiments to test their concept of evolution.

Is the topic of the opinion article discussed accurately in the context of the current literature?

Yes

Are arguments sufficiently supported by evidence from the published literature?

Yes

Are all factual statements correct and adequately supported by citations?

Yes

Are the conclusions drawn balanced and justified on the basis of the presented arguments?

Yes

Reviewer Expertise:

NA

I confirm that I have read this submission and believe that I have an appropriate level of expertise to confirm that it is of an acceptable scientific standard.

# Response

**Published:** 2020-07-21

**Authors:** Assaf Marron

**Affiliations:** Weizmann Institute of Science, Israel

We are gratified to see that Eugene Rosenberg judges the ideas presented in the paper to be original and fundamental, and that the proposed experiments are interesting and relevant.

 We thank Eugene Rosenberg for this expert review. 

 Irun R. Cohen and Assaf Marron
